# No Functional Role for microRNA-342 in a Mouse Model of Pancreatic Acinar Carcinoma

**DOI:** 10.3389/fonc.2017.00101

**Published:** 2017-05-18

**Authors:** James Dooley, Vasiliki Lagou, Emanuela Pasciuto, Michelle A. Linterman, Haydn M. Prosser, Uwe Himmelreich, Adrian Liston

**Affiliations:** ^1^Translational Immunology Laboratory, VIB, Leuven, Belgium; ^2^Department of Microbiology and Immunology, KU Leuven – University of Leuven, Leuven, Belgium; ^3^Laboratory of Lymphocyte Signaling and Development, Babraham Institute, Cambridge, UK; ^4^Wellcome Trust Sanger Institute, Wellcome Trust Genome Campus, Cambridge, UK; ^5^Department of Imaging and Pathology, KU Leuven – University of Leuven, Leuven, Belgium

**Keywords:** pancreatic cancer, microRNAs, miR-342-5p, acinar carcinoma, *in vivo*

## Abstract

The intronic microRNA (miR)-342 has been proposed as a potent tumor-suppressor gene. miR-342 is found to be downregulated or epigenetically silenced in multiple different tumor sites, and this loss of expression permits the upregulation of several key oncogenic pathways. In several different cell lines, lower miR-342 expression results in enhanced proliferation and metastasis potential, both *in vitro* and in xenogenic transplant conditions. Here, we sought to determine the function of miR-342 in an *in vivo* spontaneous cancer model, using the Ela1-TAg transgenic model of pancreatic acinar carcinoma. Through longitudinal magnetic resonance imaging monitoring of Ela1-TAg transgenic mice, either wild-type or knockout for *miR-342*, we found no role for miR-342 in the development, growth rate, or pathogenicity of pancreatic acinar carcinoma. These results indicate the importance of assessing miR function in the complex physiology of *in vivo* model systems and indicate that further functional testing of miR-342 is required before concluding it is a bona fide tumor-suppressor-miR.

## Introduction

Cancer development and growth involves the coordinated dysregulation of multiple cellular processes, including proliferation, apoptosis, migration, immune evasion, genome stability, metabolism, and angiogenesis. As global regulators capable of simultaneously regulating hundreds of genes, microRNAs (miRs) are lead candidates for coordination of the oncogenic process, with multiple miR being identified as tumor-suppressor-miR, capable of preventing oncogenesis, and oft downregulated in tumors, or onco-miR, with tumor-promoting properties and overexpression in certain tumors (1). As the technology to either supplement or suppress miR with a high degree of specificity is available and readily translated from one miR to the next, miR represent highly tempting therapeutic targets in cancer (2). Pancreatic cancer represents an important therapeutic target, with radically different expression of several miRs (3) and a distinct paucity of effective treatments leading to poor prognosis (4). It is therefore a promising line of research to functionally investigate the known miR expression changes in pancreatic cancer.

miR-342 is an intronic miR located within the host gene EVL. Both miR-342 and EVL are poorly characterized; however, miR-342 is proposed to have important oncogenic functions, with differential expression in pancreatic cancer (5) as well as acute myeloid leukemia (6, 7), breast cancer (triple-negative (8), inflammatory (9), or recurrent (10) subtypes), cervical cancer (11), colorectal cancer (12, 13), lung adenocarcinoma (14), lymphoma (15), and metastatic melanoma (16). The breadth of cancers observed to show expression changes in miR-342 suggests a common oncogenic function for these changes, mediated by altered regulation of the target mRNA. It should, however, be noted that miR-342 expression is coordinated with the host gene, EVL (17), and thus even if the observed correlation is functional it may not indicate an oncogenic role specific to miR-342.

Based on expression changes in cancer, miR-342 has been proposed as both an onco-miR and, more commonly, a tumor-suppressor-miR. During functional testing, the roles identified for miR-342 have been largely tumor-suppressing in nature, with repression of key oncogenic proteins. For example, miR-342 inhibits proliferation and invasiveness of non-small cell lung cancer cell lines after transfer into *nude* mice via regulation of RAP2B (18). Likewise, miR-342 inhibits Myc activity via regulation of E2F1 (19) and inhibits apoptosis in breast cancer cell lines via regulation of Apollon/BRUCE (20) or the human epidermal growth factor receptor 2 (HER2) pathway and inhibited the proliferation of HER2-positive cell lines (21). miR-342 has been demonstrated to inhibit the tumorigenic capacity in transplanted colon cancer cell lines, a role attributed to regulation of NAA10 (22), DNA methyltransferase 1 (23), or FOXM1 and FOXQ1 (24). The regulation of FOXM1 by miR-342 has also been proposed to be important for cervical cancer, with a function in inhibiting growth and metastasis in HeLa transplant models (25). In hepatocellular carcinoma cell lines, miR-342 inhibits proliferation by regulating the NF-κB pathway (26). Finally, miR-342 expression in the surrounding stroma may provide a tumor-suppressing function via enhancing the inhibitory effect of TGFβ on angiogenesis (27).

Together, the expression and functional data indicate miR-342 as a tumor-suppressor-miR. However, notably, to our knowledge, all functional testing has been performed in cell lines, either *in vitro* (19–21, 26) or in xenogenic transplant models (18, 22–25). Here, we sought to determine the *in vivo* functional role of miR-342 in a spontaneous murine model of pancreatic acinar carcinoma. Through longitudinal magnetic resonance imaging (MRI) assessment, we found no evidence for a tumor-suppressor role for miR-342 in the Ela1-TAg transgenic model.

## Results and Discussion

**Figure 1:**
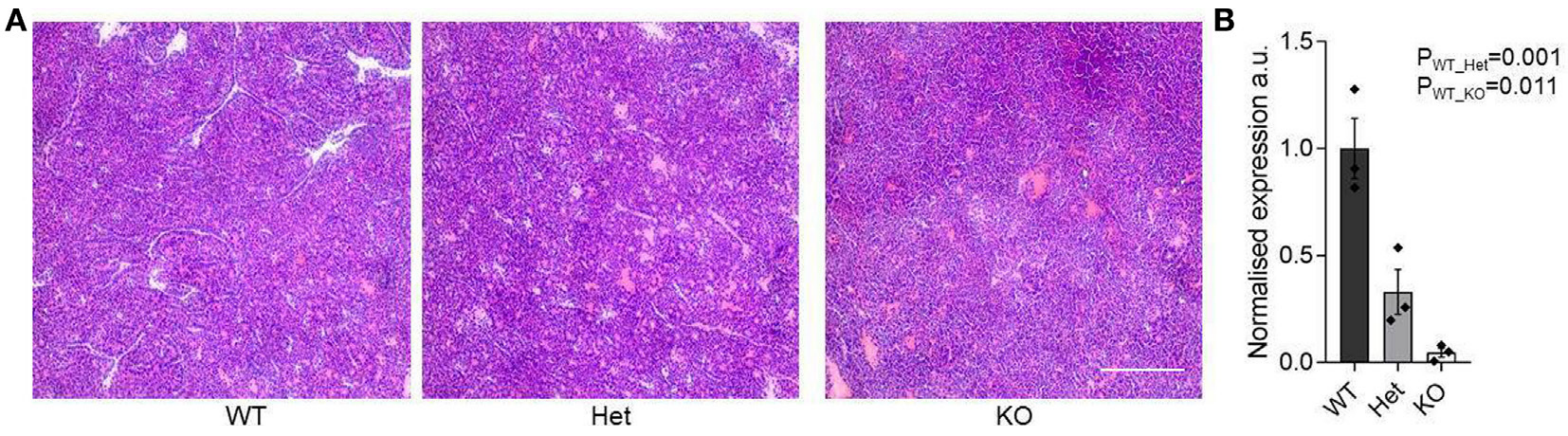
**Pancreatic acinar carcinoma development in *miR-342* knockout mice**. Pancreatic tumors were dissected from TAg^+^, *miR-342^+/0^* TAg^+^, and *miR-342^0/0^* TAg^+^ mice at 21 weeks of age. **(A)** Representative histology of tumors from each genotype. Scale = 250 μm. **(B)** Expression of miR-342 in tumors from wild-type, heterozygous, and KO mice (*n* = 3/group). Individual values shown with mean and standard error.

**Figure 2:**
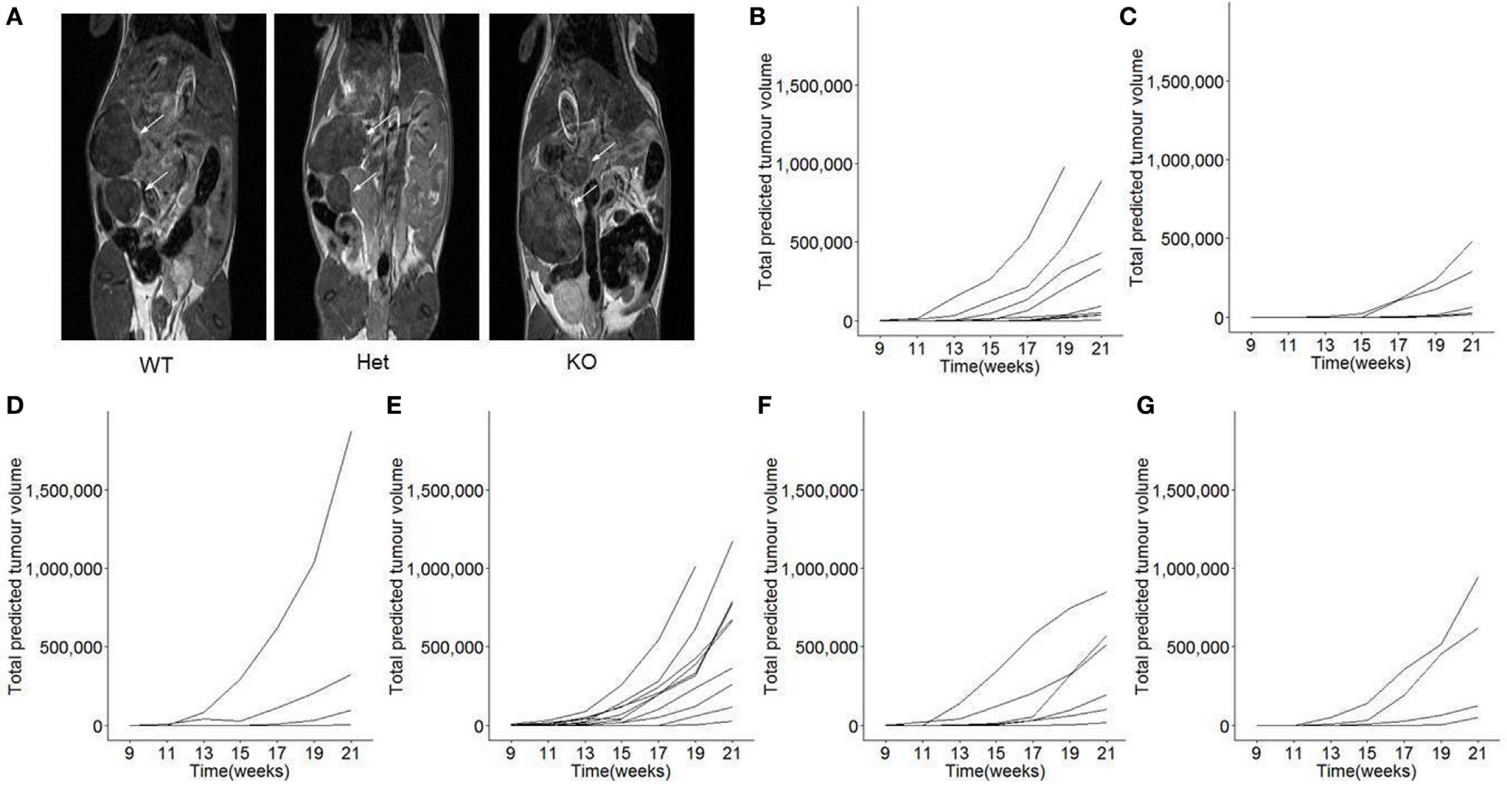
**Longitudinal monitoring of tumor growth in *miR-342* knockout mice**. TAg^+^, *miR-342^+/0^* TAg^+^, and *miR-342^0/0^* TAg^+^ mice were monitored longitudinally by magnetic resonance imaging (MRI) for tumor load and size, from 7 weeks of age until 21 weeks of age. **(A)** Representative MRI images for each genotype. **(B)** Individual total predicted tumor volume curves for TAg^+^ female mice (*n* = 10), **(C)** *miR-342^+/0^* TAg^+^ female mice (*n* = 8), **(D)** *miR-342^0/0^* TAg^+^ female mice (*n* = 6), **(E)** TAg^+^ male mice (*n* = 13), **(F)** *miR-342^+/0^* TAg^+^ male mice (*n* = 6), and **(G)** *miR-342^0/0^* TAg^+^ male mice (*n* = 4).

**Figure 3:**
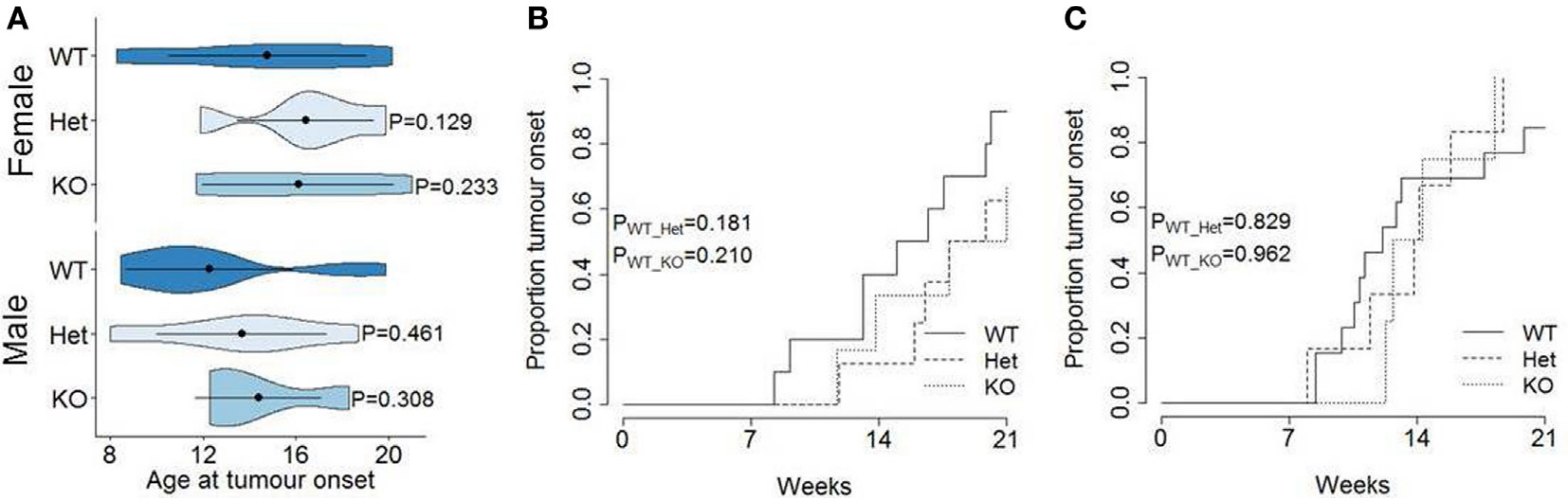
**Normal tumor onset in *miR-342* knockout mice**. TAg^+^, *miR-342^+/0^* TAg^+^, and *miR-342^0/0^* TAg^+^ mice were monitored longitudinally by magnetic resonance imaging for tumor presence. **(A)** Violin plots showing the mean, standard deviation, and kernel probability density of age at tumor onset. Cumulative incidence of pancreatic cancer in **(B)** female (*n* = 10, 8, 6) and **(C)** male (*n* = 13, 6, 4) mice.

The function of miR-342 in the development, growth, and pathogenicity of pancreatic cancer was tested by newly generating *miR-342^0/0^* mice and intercrossing with the Ela1-TAg transgenic mouse (28). The resulting *miR-342^0/0^* TAg^+^ mice, and the parental TAg^+^ strain, develop spontaneous pancreatic acinar carcinoma (Figure 1A), in which miR-342 expression was specifically lost in the KO (Figure 1B). The presence and size of tumors were assessed through longitudinal MRI assessment of tumor size from 7 weeks of age (Figure 2). Onset of cancer development was assessed in each group through analysis of first tumor detection by MRI. No significant difference in age of first tumor detection was induced by heterozygous or homozygous loss of *miR-342* (Figure 3A). This resulted in normal cumulative incidence of pancreatic cancer in both female (Figure 3B) and male (Figure 3C) mice, indicating no significant effect of miR-342 in pancreatic cancer development in this model.

**Figure 4:**
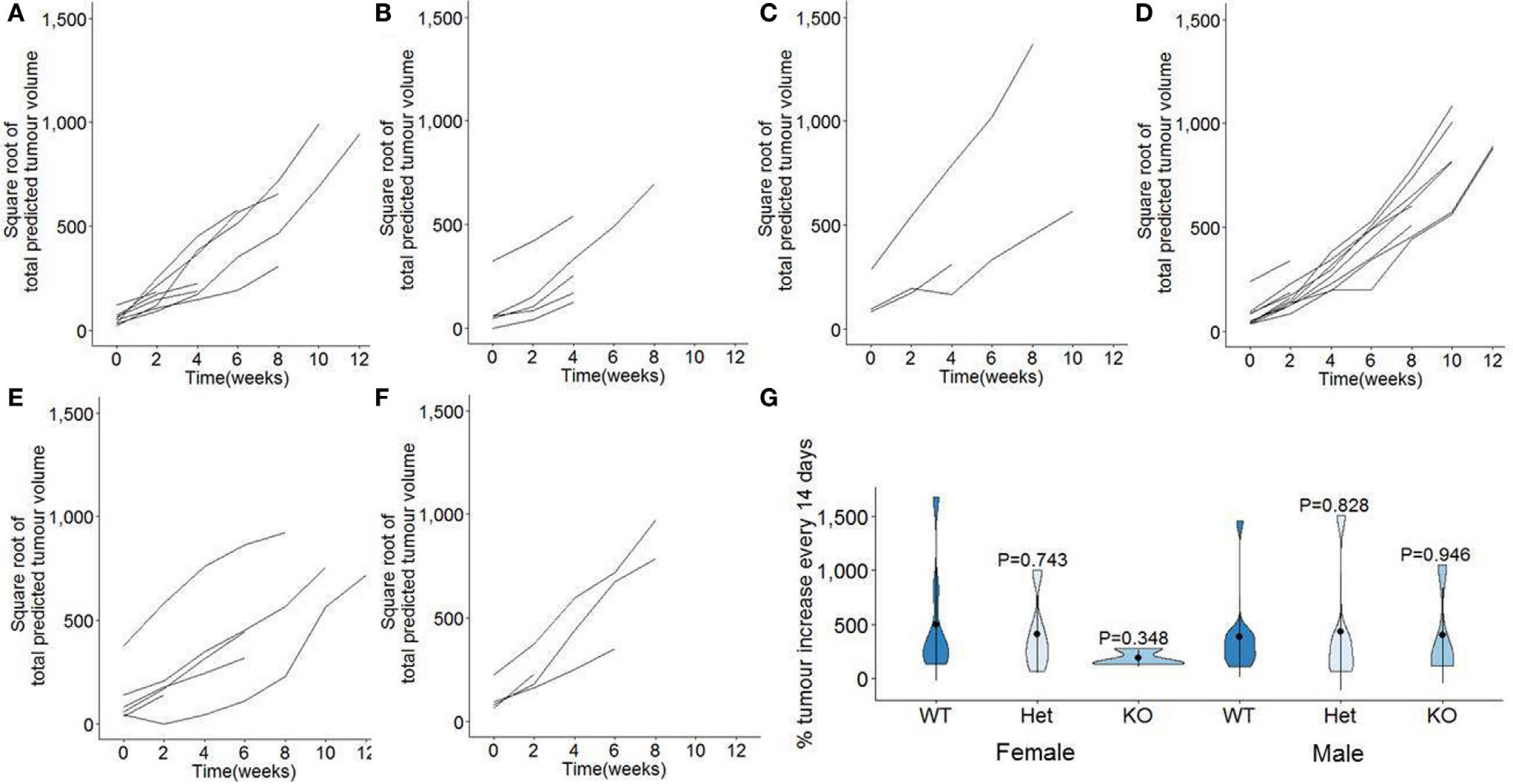
**Tumor growth rates are unaffected in *miR-342* knockout mice using a pancreatic cancer model**. TAg^+^, *miR-342^+/0^* TAg^+^, and *miR-342^0/0^* TAg^+^ mice were monitored longitudinally by magnetic resonance imaging for tumor load and size, from 7 weeks of age until 21 weeks of age. Total tumor volumes were square root transformed, with plots showing individual growth in total tumor burden from point of first detection in **(A)** TAg^+^ female mice (*n* = 10), **(B)** *miR-342^+/0^* TAg^+^ female mice (*n* = 8), **(C)** *miR-342^0/0^* TAg^+^ female mice (*n* = 6), **(D)** TAg^+^ male mice (*n* = 13), **(E)** *miR-342^+/0^* TAg^+^ male mice (*n* = 6), and **(F)** *miR-342^0/0^* TAg^+^ male mice (*n* = 4). **(G)** Violin plots for the percentage tumor volume increase per 2 weeks, averaged from time of detection to death. Mean, standard deviation, and kernel probability density.

In order to measure the function of miR-342 on the growth rates of pancreatic cancer, we normalized the longitudinal MRI assessments of tumor volume (Figure 2) to the point of first detection. Tumor volumes were square root transformed to take into account the exponential growth, providing a measurement of the growth rate observed in each individual mouse (Figures 4A–F). Growth rate curves were then calculated as the percentage of tumor volume increased observed per 14 days, averaged across the entire observation period. In miR-342-sufficient mice, tumors increased in size ±500% every 14 days, with no significant change in the growth rate observed with miR-342-deficiency, in either males or females (Figure 4G).

**Figure 5:**
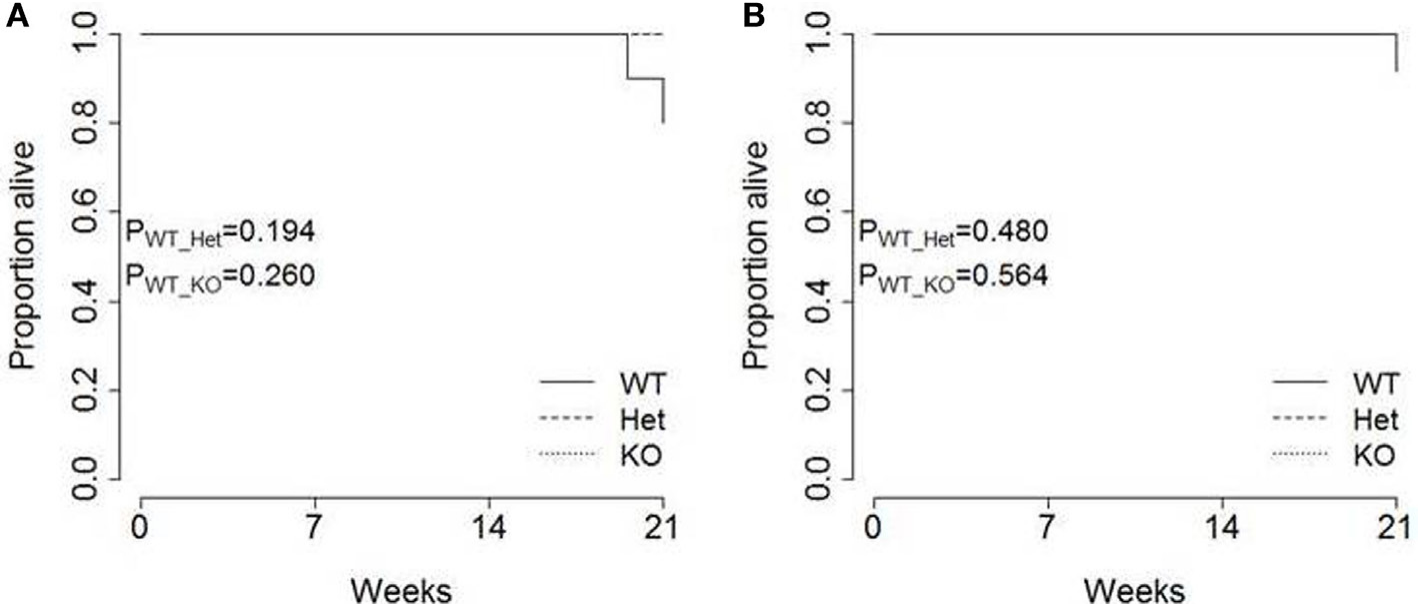
**Tumor-induced mortality is unaffected in *miR-342* knockout mice**. TAg^+^, *miR-342^+/0^* TAg^+^, and *miR-342^0/0^* TAg^+^ mice were followed until 21 weeks of age. Kaplan–Meier analysis of survival in **(A)** female (*n* = 10, 8, 6) and **(B)** male (*n* = 13, 6, 4) mice.

Finally, the pathogenic impact of pancreatic cancer was analyzed in each strain. Total survival was measured out to 21 weeks of age, with only limited mortality observed in TAg^+^ mice (Figure 5). No excess mortality was observed within this time period in *miR-342^+/0^* TAg^+^ or *miR-342^0/0^* TAg^+^ mice (Figure 5), although the limited time frame of observation does not exclude effects on survival after 21 weeks of age. Together, these results indicate that there is no major function for miR-342 in the development, growth, or pathogenicity of pancreatic cancer in the Ela1-TAg model.

While these results do not encourage the exploration of miR-342 as a clinical target in pancreatic cancer, neither do they disqualify it. The expression changes observed in miR-342 in multiple cancers may still serve as diagnostic targets with clinical benefits; indeed, expression of miR-342 has been observed to correlate with postoperative survival in pancreatic cancer (29) and response to tamoxifen treatment in breast cancer (30). Furthermore, our results, while negative, do not exclude a functional role for miR-342 in pancreatic cancer—the TAg model is just one of many increasingly sophisticated pancreatic cancer models, which more closely model the most common genetic insults observed in patient specimens (31). In addition, even the extent to which murine miR physiology mimics that of the human is unclear, with mRNA exhibiting lower levels of miR recognition conservation than protein sequence conservation (32) [although many of the predicted oncogenic targets of miR-342 are conserved from mouse to human (33)]. It therefore remains distinctly possible that further preclinical exploration will support miR-342 as a valid target for clinical intervention in a subset of pancreatic cancer patients with the appropriate molecular distortions (2).

## Materials and Methods

### Mice

The miR-342 knockout allele was generated as a 197bp targeted deletion generated in C57Bl/6N JMA.A3 mouse embryonic stem cells (34). The selection cassette was removed from the targeted allele by transient transfection of a Cre recombinase expression plasmid and the ES cells were used to generate miR-342 knockout mice in the C57Bl/6N genetic background. Ela1-Tag mice (28, 35), with transgenic expression of the SV40 large T Antigen under the Elastase-1 promoter, were purchased from Jackson on the C57BL/6 background and backcrossed to miR-342 knockout mice. Mice were bred under specific pathogen-free conditions and house under conventional conditions during MRI. All mice were fed using R/M-H ssniff chow. The study was approved by the University of Leuven Animal Ethics Committee, and all mice were used in accordance with the approved protocol. Mouse weight and blood glucose were monitored throughout the experimental process.

### Imaging

TAg^+^ mice were scanned every 2 weeks from 7 weeks of age. Mice were anesthetized using 2% isoflurane. The temperature and respiration of anesthetized mice were monitored and maintained at 37°C and >40 min^−1^, respectively. Images were acquired using a Bruker Biospin 9.4 Biospec Tesla small animal scanner (Bruker Biospin, Ettlingen, Germany) equipped with an actively shielded gradient set of 600 mT/m using a respiration triggered spin echo sequence with 50 continuous slices of 0.5 mm thickness in interlaced mode (TR = 6,000 ms, TE = 15.9 ms, FOV = 4.0 × 6.0 cm, a matrix of 200 × 400, two dummy scans, and two averages). For RF irradiation and detection, a 7.2 cm quadrature resonator (Bruker Biospin, Ettlingen) was used.

### Molecular Biology

Total RNA from pancreatic cancer tissue was extracted using Trizol Reagent (Life technologies). cDNA synthesis was performed using 20 ng total RNA and the TaqMan™ MicroRNA Reverse Transcription Kit (Applied Biosystem). Expression of mature miR-342 was determined using TaqMan™ MicroRNA Assays (hsa-miR-342-3p; Applied Biosystem). For normalization, the expression of the housekeeping genes hypoxanthine-guanine phosphoribosyltransferase and peptidyl-prolyl cis-trans isomerase A (PPIA) was measured by Syber Green real-time PCR. PCRs were performed in triplicate, and non-RT control was included.

### Statistical Analysis

Magnetic resonance imaging was analyzed using ImageJ software, and the mean area was calculated at the maximum radius. Tumor volume predictions were calculated as volume = 4/3 × area × √(area/π). Statistical analysis was made using R (https://www.r-project.org/version 3.1.2). Cumulative incidence curves were generated using the R package “survplot” with the fun = function(*x*) {1 − *x*} argument (36). Kaplan–Meier survival curves were made using the R “survplot” package. The comparison of cumulative incidence and survival distributions between two samples was performed using log-rank test implemented in the R “survdiff” package (37). Comparisons presented in the violin plots were made using two-tailed unpaired *t*-tests.

## Ethics Statement

The study was approved by the University of Leuven Animal Ethics Committee, and all mice were used in accordance with the approved protocol.

## Author Contributions

JD, ML, and AL designed the study. HP generated KO mice. JD and EP performed experiments. JD, UH, and VL analyzed the data. AL wrote the study.

## Conflict of Interest Statement

The authors declare that the research was conducted in the absence of any commercial or financial relationships that could be construed as a potential conflict of interest.
